# Supplementary material for: The interactome of intact mitochondria by cross-linking mass spectrometry provides evidence for coexisting respiratory supercomplexes
Source: Mol Cell Proteomics. 2017 Dec 8;17(2):216–32. doi: 10.1074/mcp.RA117.000470 (PMC5795388; doi:10.1074/mcp.RA117.000470)
Supplement: Supplemental Data [file supp_RA117.000470_133922_0_supp_23978_fzffwf.zip › spectra_annotation/mito_DR_spectra_annotation/17-1-6-1-6-1.pdf]

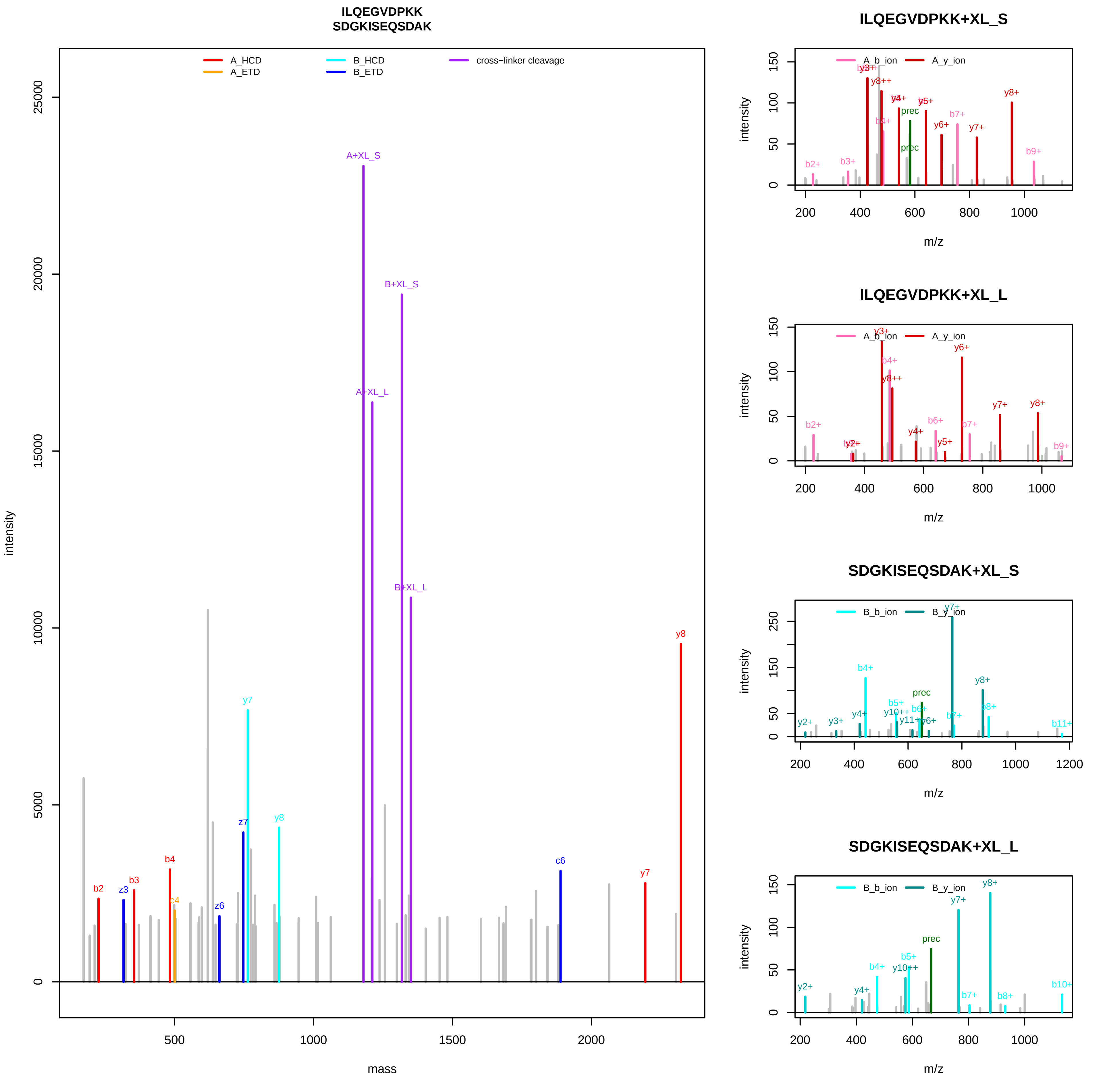
ILQEGVDPKK
SDGKISEQSDAK
A_HCD
A_ETD
B_HCD
B_ETD
cross−linker cleavage
A+XL_S
B+XL_S
A+XL_L
B+XL_L
b2
b3
b4
c4
z3
z6
z7
y7
y8
c6
y7
y8
intensity
mass
0
5000
10000
15000
20000
25000
500
1000
1500
2000
ILQEGVDPKK+XL_S
A_b_ion
A_y_ion
b2+
b3+
y3+
y8++
b4+
y4+
prec
y5+
y6+
b7+
y7+
y8+
b9+
ILQEGVDPKK+XL_L
y3+
b4+
y8++
y6+
b2+
y2+
y4+
b6+
y5+
b7+
y7+
y8+
b9+
SDGKISEQSDAK+XL_S
B_b_ion
B_y_ion
y7+
b4+
prec
y8+
b5+
y10++
b6+
y11++
y6+
b7+
b8+
y2+
y3+
y4+
b11+
SDGKISEQSDAK+XL_L
y8+
y7+
prec
b5+
y10++
b4+
y4+
y2+
b7+
b8+
b10+
intensity
m/z
